# Effects of a dietary supplement on golf drive distance and functional indices of golf performance

**DOI:** 10.1186/1550-2783-11-S1-P22

**Published:** 2014-12-01

**Authors:** Tim N Ziegenfuss, SM Habowski, RA Lemieux, JE Sandrock, AW Kedia, HL Lopez

**Affiliations:** 1 The Center for Applied Health Sciences, Stow, Ohio, USA; 2 Kent State University, Kent, Ohio, USA

## Background

Limited research is available examining the impact of different dietary supplements to impact power generation during a golf swing. Given the anaerobic nature of this task and considering the existing sports nutrition literature, it is possible that certain dietary supplements might be of benefit. The purpose of this study was to determine the impact of daily supplementation with an over-the-counter dietary supplement on golf drive distance and functional indices of golf performance.

## Methods

Twenty-seven healthy men (30.3 ± 6.9 y, 183.1 ± 5.6 cm, 86.7 ± 11.9 kg), with a handicap index of 5-15 volunteered to participate in this study and were assigned in a double-blind, placebo-controlled manner over a 30-day period to ingest either a placebo (“PLA”) or a dietary supplement containing creatine monohydrate, coffea arabica extract, calcium fructoborate and vitamin D (StrongDrive™, “SD”). All participants were assigned to ingest two doses per day of their assigned supplement for the first two weeks and one dose per day for the remaining two weeks. No exercise training or alterations in their normal physical activity habits were required/allowed and all participants were instructed to follow their normal dietary patterns. At the beginning and end of the four-week supplementation period, participants completed two identical testing sessions consisting of a fasting blood sample, anthropometric measurements, 1RM bench press, upper body power (via 3 sets of bench press throws) and a series of 10 successive golf swings (using their driver and 7-iron) that were analyzed three-dimensionally for changes in indices of golf performance. Diet records, quality of life, pain inventories and adverse events were also collected at the beginning and end of the study. Data were analyzed using separate ANOVA and ANCOVA with respective baseline scores as the covariate, respectively. Within-group changes were also probed with paired samples t-tests and effect size (ES) calculations. Statistical significance was established a priori at p<0.05. Consent to publish the results was obtained from all participants.

## Results

ANCOVA revealed a significantly greater (post-test) best drive distance for SD (+5.0% [+13.6 yards], p=0.04, ES = 0.75) as well as a tendency for average drive distance to increase (+8.4% [+19.6 yards], p=0.07, ES = 0.65), while no such changes were found with the PLA group (-0.5% [-1.2 yards], p=0.82, ES = 0.04 and +1.3% [+2.8 yards], p=0.70, ES = 0.08, respectively). Both groups experienced significant increases in body mass and 1RM bench press (p<0.001). A significant group x time interaction was found for average upper body velocity during the 1st set of bench throws (p=0.02) while peak velocity tended to change (p=0.07). Within-group analysis confirmed significant improvements in average velocity (+8.9%, p=0.001) and peak velocity (+6.8%, p=0.005) for SD only. No changes were noted for reported adverse events, pain inventories, quality of life or any components of the complete blood count, comprehensive metabolic panels, c-reactive protein or vitamin D (p>0.05).

## Conclusion

The use of SD for 30 days appears to favorably impact changes in golf drive distance and upper body power/performance. Supplementation was well tolerated and did not result in any clinically significant changes in markers of health or adverse events/side effect profiles. Future studies are being planned to continue exploring the nature/strength of this association, its potential mechanisms, and other aspects of golf performance that may be impacted by SD supplementation.

